# Heart Rate and Heart Rate Variability during Sleep in Family Dogs (*Canis familiaris*). Moderate Effect of Pre-Sleep Emotions

**DOI:** 10.3390/ani8070107

**Published:** 2018-07-02

**Authors:** Bence Varga, Anna Gergely, Ágoston Galambos, Anna Kis

**Affiliations:** 1 Institute of Cognitive Neuroscience and Psychology, Hungarian Academy of Sciences, 1117 Budapest, Hungary; bencevargaa@gmail.com (B.V.); anna.gergely66@yahoo.com (A.G.); hankie.moody@gmail.com (Á.G.); 2 Department of Cognitive Science, Budapest University of Technology and Economics, 1111 Budapest, Hungary; 3 Department of Cognitive Psychology, Eötvös Loránd University, 1053 Budapest, Hungary

**Keywords:** dog, sleep, heart rate, emotion

## Abstract

**Simple Summary:**

It is common knowledge that negative emotions in humans are accompanied by both impaired subjective experience as well as maladaptive changes in behavior and physiology. The present paper investigates heart rate—one of the most commonly used emotion-related physiology measures—in the family dog, with the aim of uncovering its potential relationship with emotions. Sleep recordings were conducted following a positive versus a negative social interaction, as sleep alternations are one of the most conspicuous changes in response to negative affect. We observed differences in heart rate following the positive versus negative interactions, however these were only apparent during wakefulness, but not during sleep.

**Abstract:**

The domestic dog (*Canis familiaris*) has been shown to both excel in recognising human emotions and produce emotion-related vocalisations and postures that humans can easily recognise. However, little is known about the effect of emotional experiences on subsequent sleep physiology, a set of phenomena heavily interrelated with emotions in the case of humans. The present paper examines heart rate (HR) and heart rate variability (HRV) during dogs’ sleep, measures that are influenced by both positive and negative emotions in awake dogs. In Study I, descriptive HR and HRV data is provided on *N* = 12 dogs about the different sleep stages (wake, drowsiness, non-rapid eye movement (non-REM), REM; scoring based on electroencephalogram (EEG) data). We conclude that wakefulness is characterised by higher HR and lower HRV compared to all sleep stages. Furthermore, drowsiness is characterised by higher HR and lower HRV than non-REM and REM, but only if the electrocardiogram (ECG) samples are taken from the first occurrence of a given sleep stage, not when the longest periods of each sleep stage are analysed. Non-REM and REM sleep were not found to be different from each other in either HR or HRV parameters. In Study II, sleep HR and HRV measures are compared in *N* = 16 dogs after a positive versus negative social interaction (within-subject design). The positive social interaction consisted of petting and ball play, while the negative social interaction was a mixture of separation, threatening approach and still face test. Results are consistent with the two-dimensional emotion hypothesis in that following the intense positive interaction more elevated HR and decreased HRV is found compared to the mildly negative (lower intensity) interaction. However, although this trend can be observed in all sleep stages except for REM, the results only reach significance in the wake stage. In sum, the present findings suggest that HR and HRV are possible to measure during dogs’ sleep, and can potentially be used to study the effect of emotions not only during but also after such interactions.

## 1. Introduction

Despite the existence of some fish and amphibian species that do not show any apparent behavioural signs of sleep [1], it is widely accepted that sleep is an evolutionarily adaptive phenomenon, which has crucial importance for daily normal functioning. The importance of sleep is not only salient from the consequences of sleep deprivation (including hypertension, obesity or diabetes [2]), but also from its link with cognitive (e.g., memory consolidation [3,4]) and affective [5] functioning. The present study builds on results from this latter direction of research and explores in family dogs heart rate and heart rate variability measures during sleep preceded by positively versus negatively valenced social interactions.

Sleep can be divided into two distinct stages: rapid eye movement (REM) and non-rapid eye movement (non-REM) sleep. Non-REM sleep is also often called slow wave sleep, due to the apparent low frequency, high amplitude electroencephalogram (EEG) activity during the “deep sleep” part of this stage. REM sleep is also known as paradoxical sleep due to characteristic high frequency, low amplitude EEG activity paired with muscle atonia. Beside EEG, the activity of the autonomic nervous system (ANS) as indicated by heart rate (HR) and heart rate variability (HRV) are frequently used as physiological correlates of the level of activation along the sleep-wake axis [6]. During a sleep cycle non-REM and REM sleep are modulated differently by the ANS. After sleep onset parasympathetic activity increases, resulting in a lower HR and higher HRV [7]. This parasympathetic dominance prevails during the non-REM phase (with sympathetic activity reduced), while the REM phase is characterised by fluctuations in parasympathetic and sympathetic control [8].

Heart rate has been used as an indicator of the physiological state in both animals and humans [9], and it is generally assumed that increased levels of stress are reflected in increased HR. Due to the autonomic innervations of the heart, HRV can be a good physiological index of emotion regulation, since the central autonomic network (CAN) uses brain areas that are also involved in emotion regulation, like the anterior cingulum, ventral medial prefrontal cortex or the central nucleus of the amygdala [10,11]. In addition, CAN provides the parasympathetic fibres to the heart, therefore HRV can be a good indirect measure of physiological capacity for emotion regulation [12].

Emotional experiences during the day can alter sleep quality and macrostructure in many ways [13]. For example REM sleep has been reported to be enhanced [14], shortened [15] or unchanged due to different stressors. More fragmented sleep, longer sleep latency, shorter sleep duration also frequently occurs following stressful events [16]. Insufficient and/or disturbed sleep (sleep deprivation), in turn, alters mood and emotion regulation [17,18,19]. It appears that sleep deprivation diminishes the top–down control of the prefrontal cortex over the amygdala (the main structure in the processing of emotion-related information, especially aversive stimuli [20]), and activation of the amygdala increases in response to negative pictures [17].

While both the relationship between (awake) HR and emotions, as well as sleep and emotions are relatively well-studied separately, little is known about how HR and HRV change during sleep following an emotionally loaded event. The current paper will investigate this question in family dogs (*Canis familiaris*), the most common pet animal around the world, that experience natural situations inducing both positive and negative affect in our anthropocentric environment. Dogs have been widely used as models of human behaviour due to the fact that during domestication this species has adapted to the same environmental challenges as humans [21,22,23] and has thus developed several human-like socio-cognitive skills [24,25]. This makes them further valuable as a model system, since due to the increasing scientific interest in the species [26], considerable amount of information has accumulated on their behaviour and neurocognition [27]. Extensive data exists about dogs’ emotional processing, for example showing that they spontaneously distinguish between human emotions based on facial expressions [28,29,30,31]. Furthermore, dogs are able to base their in-test choices on human emotional expressions [32,33]. Dogs have also been shown to experience environmental stress [34,35], and they may develop phobias (e.g., thunderstorm-phobia [36]). Some argue that dogs even possess secondary emotions like jealousy [37] and guilt [38,39].

Several studies have found a relationship between dogs’ HR responses and stress in different situations [40,41,42]. On the other hand, dogs’ HR and HRV have also been linked to positive emotions [43,44]. Research with shelter dogs has found that human-animal interactions not only alter HRV, but the magnitude of the HRV response is related to behavioural data [45].

Recent studies also started to address the sleep physiology of family dogs, with a special interest in its relationship to dogs’ human-analogue social behaviours. It has been shown that dogs, despite displaying a polyphasic sleep structure, show several similarities to human sleep including the EEG spectra of the different sleep stages [46], as well as the interrelatedness of sleep and memory consolidation [47,48]. Furthermore, recently it was found that significant differences can be observed in the macrostructure of sleep following negative (threatening approach, separation) or positive (playing, petting) pre-treatments [49]. Data from these non-invasive studies is mostly in line with the decades of canine sleep research carried out with invasive methods, mainly focused on neurological conditions such as epilepsy [50] and narcolepsy [51]. Dogs are known to display polyphasic sleep [52] with short sleep–wake cycles [53]. A clear circadian, diurnal rhythm was also found in dogs [54] similar to that in humans, and it was reported that dogs are most active after light onset and tend to have a rest during the afternoon [55].

The present study capitalizes on these recent findings and explores the effect of positively versus negatively valenced social interactions on heart rate and heart rate variability during different stages of subsequent sleep in dogs. In order to do so, we first conduct a methodological study to establish heart rate measurements during sleep in dogs. Thus, Study I will provide descriptive data on dogs’ heart rate and heart rate variability during different stages of sleep after testing the effect of sampling length (20 s, 1 min, 5 min segments used for calculating heart rate data) as well as sample selection (longest period of the given sleep stage versus first occurrence of a given sleep stage). This is followed by Study II, testing the effect of pre-sleep positive and negative social interactions by analysing the ECG data from polysomnography recordings by [49].

## 2. Study I

### 2.1. Background

Due to inconsistency in the literature as to the sample lengths used for ECG analysis (e.g., using 30 s intervals in every 10 min for 24 h [56], averaging a whole 48 h recording [54]) our first aim was to test the potential effect of such differences. In case of humans, the 5 min recording seems to be the most appropriate interval [57], while dog studies have often used 1 min sampling intervals. Thus, we decided to use both these sample lengths, as well as a shorter (20 s) interval in order to see if reliable results can be obtained with less effort or if longer samples are indeed needed. Since manual detection of the RR peaks in the ECG recording (due to the sinus arrhythmia that characterizes dog heart rate, automatic measures are hard to apply [58]) is labour-intensive, reducing the sampling interval without compromising data reliability would be highly advantageous.

Polysomnography recordings typically last several hours, and both sleep macrostructure as well as EEG spectrum are known to change over time, which is also the case in dogs [59]. Thus, a further important question for sleep ECG measures on short time intervals is the timing of the samples selected for analyses. Two equally logical options would be to take either the first occurrence of a given stage or the longest period of the given stage as most representative. As no data is available in the literature about changes in canine ECG during successive sleep cycles, we have decided to do our analyses with both of these sampling methods. Thus, comparisons of the different sleep stages (wake, drowsiness, non-REM, REM) will be provided both based on the longest period of the given stage and on the first occurrence of the given stage.

### 2.2. Methods

#### 2.2.1. Subjects

The subjects for Study I were 12 adult (mean age ± SE: 4.17 ± 3.01 years, range: 1 to 10 years) pet dogs (6 male and 6 female) from seven different breeds (4 Golden Retrievers, 1 Border Collie, 1 Malinois Mix, 1 Cain Terrier, 1 Hungarian Vizsla, 1 Shetland Sheepdog, 1 German Shepherd) and 2 mongrels. All dogs were older than 1 year of age and were not trained for the examinations.

#### 2.2.2. General Procedure

The measurements took place in a room equipped with a mattress on the floor for the owner and the dog, a table lamp for the owner to read during the test and a water bowl for the dog. Upon arrival, the subject had approximately 10 min to explore the environment, then the experimental preparations started.

A 3 h non-invasive polysomnography measurement (see Supplementary Materials for details) was conducted with each dog, which followed a validated protocol [46] and allowed us to record spontaneous natural sleep (no sedatives were used). For the present study the electrocardiogram (ECG) signal was analyzed, recorded with two electrodes placed bilaterally on the second ribs. Gold coated Ag|AgCl electrodes were used, attached with EC2 Grass Electrode Cream (Grass Technologies, Madison, WI, USA). Signals were collected, prefiltered, amplified and digitized at a sampling rate of 249 Hz/channel by using the 30-channel Flat Style SLEEP La Mont Headbox with implemented second order filters at 0.5 Hz (high pass) and 70 Hz (lowpass) as well as the HBX32-SLP 32 channel preamplifier (La Mont Medical Inc., Madison, WI, USA). After these preparations, we left the dog alone in the room with its owner, who was allowed to watch a film (with headphones), learn or read in silence in order to let the dog sleep.

#### 2.2.3. Data Analysis

For all recordings sleep macrostructure was scored according to previously validated standard criteria [46] in 20 s epochs, which identified four stages: wake, drowsiness, non-REM, and REM.

**Figure 1:**
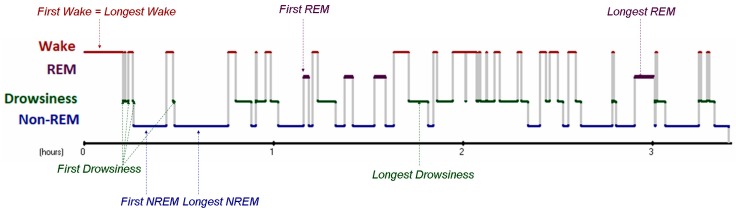
Sample hypnogram showing the sleep structure of one subject, with the arrows indicating the periods selected for electrocardiogram (ECG) analysis from all sleep stages. REM: rapid eye movement; non-REM/NREM: non-rapid eye movement.

Using the ECG channel, R peaks were manually detected on pre-defined time intervals, and RR intervals were measured using the Fercio’s software (©Ferenc Gombos 2009–2016). From all sleep stages (wake, drowsiness, non-REM, REM) the first occurrence of the given stage as well as the longest period available was selected (Figure 1) for analysis. From each such period RR intervals from a 20 s, a 1 min and a 5 min interval were used for calculating HR and HRV measures (see later). For each analysis one sample/dog was used. Note that the first period of a given sleep stage might coincide with the longest period. Furthermore, when selecting the first occurrence of a given sleep stage, this period might be shorter than 5 min, thus the 5 min interval analysed often composed of multiple shorter episodes.

From these RR interval data, heart rate, as well as time domain-based heart rate variability parameters were calculated. These parameters were the following: HR (heart rate, 60/mean RR, the number of heart beats per minute), SDRR (the standard deviation of the RR intervals), meanSD20secRR (the RR intervals mean standard deviation in the epochs (20 s), rMSSD (the root Mean Square of Successful Differences), RR50 count (the number of subsequent RR intervals that differ by more than 50 ms), pRR50 (percentage of the RR50 count on a given time series). These parameters were used to compare sampling interval length (20 s, 1 min, 5 min), and the different sleep stages (wake, drowsiness, non-REM, REM), as well as to assess the effect of sampling method (first occurrence versus longest period).

#### 2.2.4. Statistical Analysis

First, the effect of sample length (20 s versus 1 min versus 5 min) was tested using the longest occurrence of all sleep stages with paired *t*-tests for all possible combinations. False discovery rate adjustment of significance levels for multiple comparison were applied here and in all subsequent analysis according to previously described criteria [60].

Second, the four sleep stages (wake versus drowsiness versus non-REM versus REM) were compared for each ECG parameter using 5 min samples from the longest occurrence of the given sleep stage (Friedman test followed by paired t-tests in case of significant effect).

Third, the above analyses comparing the 20 s versus 1 min versus 5 min sampling intervals (paired t-tests on all sleep stages) as well as comparing the four sleep stages (5 min sampling interval; Friedman test, paired *t*-tests) was repeated using the first occurrence of the given stage.

### 2.3. Results

Differences were found in several parameters between the three sampling times (20 sec, 1 min, 5 min; Table 1), suggesting that a 5 min long sampling interval might be preferable as opposed to the commonly used 1 min. Specifically, 5 min measures of mSD20sRR in drowsiness (t_(11)_ = 2.426; *p* = 0.034) and of SDRR in non-REM (t_(11)_ = 3.252; *p* = 0.008) were different from respective 1 min measures. Comparison of 5 min versus 20 s also differed in two cases (drowsiness, rMSSD: t_(11)_ = 2.769; *p* = 0.018; non-REM, pRR50: t_(11)_ = −2.827, *p* = 0.016). Further differences occurred in 1 min versus 20 s (HR, wakefulness: t_(11)_ = 2.279; *p* = 0.044; HR non-REM: t_(11)_ = 2.555; *p* = 0.027 and pRR50, non-REM: t_(11)_ = −2.402; *p* = 0.035). The RR50count parameter was not included in this analysis, as it is inherently sampling time-sensitive. Based on this result, the following analyses were conducted using 5 min sampling intervals.

Comparing the four sleep stages (wake, drowsiness, non-REM, REM), differences were found in five out of the six ECG parameters (Table 2). Notably, the wake phase differed from all other sleep stages (drowsiness, non-REM, REM) showing a higher HR and a lower HRV (SDRR, mSD20secRR, RMSSD, pRR50, but not RR50 count). However, the sleep stages did not differ from each other, except for the pRR50 parameter which was lower in REM sleep compared to non-REM sleep (with the values for drowsiness being in between, not different from either of the two).

Using a different sampling method (selecting the first occurrence of a given stage) also resulted in differences between 5 min versus 1 min versus 20 s sampling intervals (Table 3). Two such differences were the same as with the previous sampling method (selecting the longest period of the given stage): SDRR during non-REM (5 min versus 1 min: t_(11)_ = 2.780; *p* = 0.018), mSD20sRR during drowsiness (5 min versus 1 min: t_(11)_ = 2.235; *p* = 0.047). Further differences were also found: HR during drowsiness (5 min versus 1 min: t_(11)_ = 3.148; *p* = 0.009), SDRR during drowsiness (5 min versus 1 min: t_(11)_ = 2.493; *p* = 0.030), mSD20sRR during non-REM (5 min versus 1 min: t_(11)_ = 2.887; *p* = 0.015). All other contrasts were non-significant.

**Figure 2:**
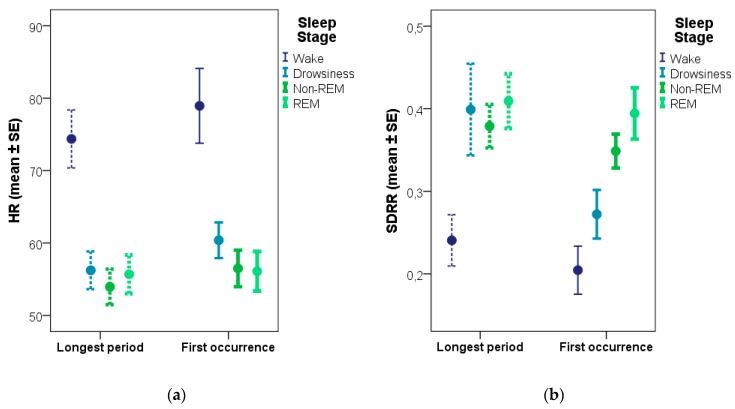
Differences between sleep and awake stages based on the longest period versus the first occurrence of the given stage regarding heart rate (**a**) and SDRR, a heart rate variability measure (**b**).

Comparing the four sleep stages (wake, drowsiness, non-REM, REM), differences were found in the same five (out of the six) ECG parameters as in case of the analysis using the longest period of the given stage (Table 4). However, in addition to the above revealed differences between the wake stage and sleep (drowsiness, non-REM, REM), this analysis also revealed differences contrasting drowsiness with the other two sleep stages (non-REM, REM). This analysis again showed that non-REM and REM sleep are characterized by similar ECG values, only two non-significant differences were found regarding SDRR and pRR50% (with the latter trend also being present in the analysis based on the longest periods). HR and one of the HRV measures (SDRR) of the different sleep stages for both sampling methods are shown on Figure 2.

## 3. Study II

### 3.1. Background

HRV is a promising measure to assess emotional states in dogs [61,62], including these events’ impact on sleep (see examples for humans [63,64], or dogs [35,36]). Thus, for Study II, we analysed the ECG channel of polysomnography recordings conducted after a short positive versus a negative dog–human social interaction (see later). The original study [49] found that sleep macrostructure was markedly different between pre-treatment conditions, with a shorter sleep latency after the negative social interaction and a redistribution of the time spent in the different sleep stages. However, changes in cardiac activity have not been investigated in this context. The controlled interactions used in that study for both the positive and the negative condition had been previously reported to influence on-line ECG measures. During separation from the owner, dogs’ HR was found to increase (despite dogs’ decreased activity) in one study [65] while in another study [61] HRV significantly increased in the same separation situation. The threatening approach of an experimenter was found to increase dogs’ mean HR, with a parallel decrease in the HRV [61]. On the other hand, petting of a dog by an experimenter was found to produce deceleration in HR in most dogs [66,67]. However, it is not yet known if the effect of such short interactions would also manifest during subsequent sleep.

### 3.2. Methods

#### 3.2.1. Subjects

A different group of *N* = 14 adult (mean age ± SE: 4.92 ± 0.91 years, range: 1−12 years) pet dogs (7 males, 7 females) from nine different breeds (2 Hungarian Vizslas, 2 Hovawarts, 1 Golden Retriever, 1 Labrador Retriever, 1 Mudi, 1 Shetland Sheepdog, 1 Belgian Shepherd (Groenendael), 1 Boxer, 1 Jack Russell Terrier) and 3 mongrels participated in Study II. There were no specific requirements for participation except that dogs had to be older than 1 year. Dogs were not trained for the purpose of this experiment. (Note that originally *N* = 16 dogs participated in this experiment [49], however the ECG signal could not be analysed in case of 2 subjects due to artefacts impeding R peak detection.)

#### 3.2.2. Procedure

All of our subjects participated in 3 h long polysomnography recordings on a total of three occasions following the above described non-invasive protocol but using a different recording system. Signals were collected, pre-filtered, amplified and digitized at a sampling rate of 1024 Hz/channel, by using the 25 channel SAM 25R EEG System (Micromed, Mogliano Veneto, Italy), as well as the System Plus Evolution software with second-order filters at 0.016 Hz (high pass) and 70 Hz (low pass).

The first polysomnography occasion was an adaptation session and thus was not analysed subsequently, but merely aimed to familiarize the subjects with the laboratory, the experimenter and the electrode placement. The second and third polysomnography recordings were preceded by a socially positive or negative pre-treatment of 6 min duration, respectively (within-subject design, counterbalanced treatment order, so that eight randomly chosen dogs participated first in the positive treatment and second in the negative treatment, while other eight dogs participated in the reverse order). During the positive social interaction (PSI) dogs had the opportunity to play with the owner and the unfamiliar experimenter (tug of war and/or throw and fetch depending on the dog’s preference) and both the experimenter and the owner petted the dog every time the dog approached them and used dog-directed speech towards the subject. The negative social interaction (NSI) consisted of a separation episode when the dog was left alone (2 min), a threatening approach by an unfamiliar experimenter (1 min), and a still face situation (3 min). The sleep structure data following the PSI and NSI was reported in [49], see also that paper for further procedural details. The behaviour of the dogs during PSI and NSI was also reported in [49], with dogs during PSI receiving petting from and engaging in play with both the owner and the experimenter, while during NSI looking and standing at the door in separation, moving off to avoid the threatening human and averting gaze and vocalising during the sill face test.

For the current analysis the ECG measures described in Study I (HR, SDRR, mSD20secRR, RMSSD, RR50count, pRR50) were used to find differences between the PSI and NSI conditions. Based on the above results, 5 min long ECG samples were taken from the first occurrence of each sleep stage.

#### 3.2.3. Statistical Analysis

Each ECG measure (HR, SDRR, mSD20s, RMSSD, RR50c, pRR50%) was compared between recordings following the PSI versus NSI separately for the four sleep stages (wake, drowsiness, non-REM, REM) using a 5 min sampling interval from the first occurrence of the given stage (paired *t*-tests, false discovery rate adjustment). In addition, individual consistency for each ECG measure was tested by partial correlations run on the two data points recoded for each sleep stage controlling for sequence of interactions (PSI–NSI vs. NSI–PSI).

### 3.3. Results

**Figure 3:**
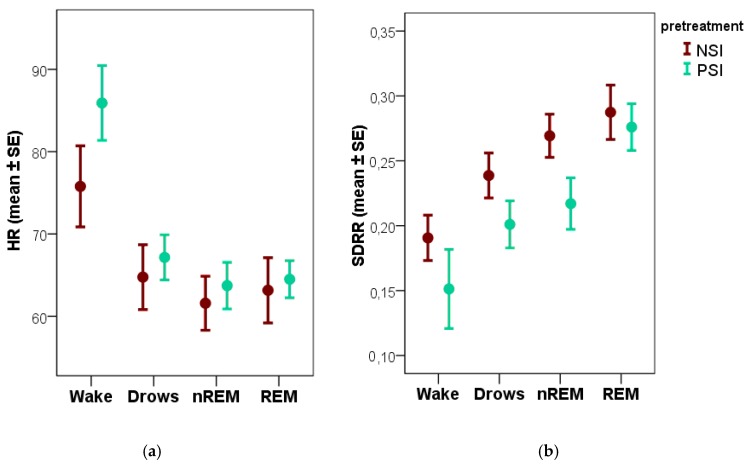
Differences in heart rate (**a**) and SDRR, a heart rate variability measure (**b**) during wakefulness and the sleep stages following positive versus negative social interaction. NSI: negative social interaction; PSI: positive social interaction.

Dogs’ heart rate and heart rate variability differed between the recordings following the positive versus negative social interaction in several parameters. However, all of the differences that remained significant after false discovery rate adjustment were in the wake phase (Table 5). During the wake phase HR was higher, while the HRV measures (mSD20s, RMSSD and pRR50%) were lower following PSI compared to NSI (the same direction was observed, but non-significant for RR50c, while SDRR did not differ between conditions). Trends in the HR and HRV measures during non-REM followed the same direction, but none of these effects reached significance. No differences were observed between conditions during drowsiness and REM. HR and one of the HRV measures (SDRR) of the different sleep stages for both PSI and NSI are shown on Figure 3.

**Figure 4:**
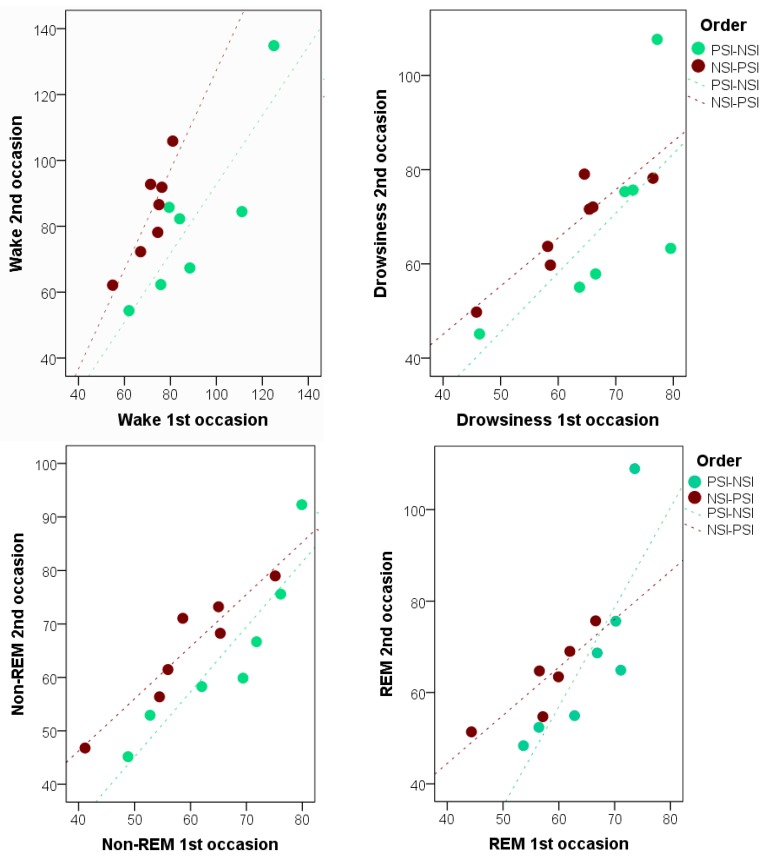
Relationship between heart rate during the first versus the second occasion in dogs staring with PSI and dogs starting with NSI (5 min sampling from the first occurrence of wakefulness, drowsiness, non-REM and REM).

The analysis of individual consistency on HR and HRV parameters in the different sleep stages revealed several significant within-individual correlations (Table 6). Individuals with higher HR on one occasion would also show higher HR on the other occasion in all sleep stages (all r > 0.7, all *p* < 0.005). Most individual consistency was observed in the REM phase, where apart from HR, several HRV measures (SDRR, RMSSD, RR50count) also showed significant individual consistency. However, in all sleep stages at least one HRV parameter was found that showed significant individual consistency. HR data for the four stages (wake, drowsiness, non-REM, REM) is plotted on Figure 4.

## 4. Discussion

This study was the first to quantitatively describe heart rate and heart rate variability during different stages of sleep in dogs. Our results show that there is a clear differentiation between ECG parameters during wakefulness versus sleep, while the differences between the distinct sleep stages are less pronounced. This finding is somewhat surprising given the fact that dogs’ sleep includes a so called drowsiness stage [68,69] that, based on the EEG signal, acts like a transition between sleep and wake. Since the analysis of the first occurrence of each sleep stage (as opposed to the longest period of the given stage) found differences between not only wake and sleep, but also between drowsiness and sleep (non-REM, REM), we believe this sampling method to be the more reliable of the two.

Comparing our data to the literature, similar heart rate values have been found for resting and awake dogs, as in our study (74.4 and 78.9 bpm for the longest and the first wake period respectively). A study comparing dogs’ HR in different postures [70] found approximately 90 bpm for lying, which overlaps with results [71] from a group of resting (awake, un-anesthetized, lying) dogs that had a HR of 54–90 bpm, and is almost identical to a previous study from our group [72] that found a mean ± SE HR of 89.47 ± 6.67 in resting awake dogs. Data from the current study are somewhat lower compared to the 90 bmp, which might suggest that dogs shortly before falling asleep have already lowered HR compared to simply lying down for a few minutes. Regarding HR during sleep, in studies using 24 h monitoring (without EEG-based sleep stage scoring) the minimum HR values fall close to our measures during both REM (55.7 bpm and 56.1 bpm) and non-REM (54.2 and 56.1 bpm): minimum mean 24 h heart rate (during behavioural sleep) was 43.12 ± 10.96 bpm in one study [73], while in another study [74] the 1 h periods with the lowest HR had 61 ± 3.5 bmp (4:00–5:00) and 62 ± 4.0 bmp (2:00–3:00). These data, together with our results following the expected tendencies between sleep stages (highest HR in wake, followed by drowsiness and then non-REM and REM sleep) [8] are indicative of the validity of the methods used in the current study. The expected difference between drowsiness and the other sleep stages (non-REM, REM) is only apparent when contrasting samples from the first occurrence of a given stage (but not with samples from the longest periods) which might indicate that the validity of the ECG measures is more heavily compromised by using samples that are further away in time.

Comparing the three sampling lengths (20 s, 1 min and 5 min) some differences were found in the ECG measures suggesting that the 5 min sampling is necessary for properly measuring the HRV changes. Previous studies have often used 1 min samples with both dogs [75] and humans [76,77] or even only a few seconds long recordings [78]. Our current finding is in line with the outcome of a former human study that claimed the 5 min HRV results to be stable enough over time [57].

Cardiac change is often used as a general psycho-physiological indicator, including in several domestic species [62,79,80]. Our results add to this literature by showing that changes in dogs’ HR and HRV are also noticeable after the positively versus negatively valenced social interactions. We should note, however, that such differences were only apparent during wakefulness, which is the very first stage in our recordings. Further studies should investigate if the non-significant trends found during non-REM sleep are indeed caused by the positive and negative social interactions that preceded the recordings.

Our results show that (during the wake phase) HR was higher, while the HRV measures were lower following the positive compared to the negative social interaction. This direction of change is contrary to what has been found in case of online ECG analysis, where negative interactions such as separation and threatening approach increased HR and decreased HRV [65], while positive interactions such as petting by a human decreased HR [66,67]. Findings of the current study are instead more in line with the notion of the two-dimensional emotion model [81] according to which not only valence (positivity/negativity), but also the arousal (low to high ‘activation’ or ‘intensity’) determines a given emotional experience. Based on the predictions of this model, intensity-dependent measures would react to a bigger extent to more intense emotions regardless of the valence (positivity or negativity) of the situation. There are some indications in the literature that (awake) heart rate data is one such intensity-dependent measure [82]. Since due to ethical considerations the present study used an intense positive treatment, while only a mildly stressful negative treatment, the response pattern we found follows that of an intensity-dependent measure. This does not invalidate the previous finding, as low-key petting used as positive stimuli [66] has been found to increase endogenous oxytocin release in dogs [83], while intranasal oxytocin treatment, in turn, decreases dogs’ HR and increases HRV [84]. In order to disentangle all possible hypotheses proposed by the two-dimensional emotion model, more research will be needed with positive and negative treatments of different intensity as well as a neutral baseline.

In addition, strong individual consistency was found between occasions in Study II. This is in line with the literature suggesting that several individual characteristics including age [85], gender [86], body mass [87] and behavioural traits such as personality [88] influence ECG parameters. It is unclear why in the current study some parameters showed within-individual consistency in certain sleep stages, while others did not, but the fact that several such within-individual correlations were found serves as a further methodological validation. Furthermore, this strong individual consistency together with the observed (and expected [70]) high individual variation might have masked in some cases the effect of positive and negative emotional treatments, suggesting that studying more homogenous populations might be necessary in the future.

## 5. Conclusions

In summary, our results provide the first experimental examination of dogs’ ECG parameters during different stages of sleep. Beside describing differences between sleep and awake stages, we also highlight important methodological considerations such as the timing of the sample and sampling length, as well as provide evidence for the effect of positive and negative social interactions on HR and HRV after the treatment.

## Figures and Tables

**Table 1:** Summary of results comparing the different sampling intervals from the longest occurrence of all sleep stages. The direction of significant differences are indicated with inequality signs (>, <). HR: heart rate; SDRR: the standard deviation of the RR intervals; meanSD20secRR: the RR intervals mean standard deviation in the epochs (20 s), rMSSD: the root Mean Square of Successful Differences; RR50 count: the number of subsequent RR intervals that differ by more than 50 ms. pRR50: percentage of the RR50 count on a given time series.

	HR	SDRR	mSD20sRR	rMSSD	pRR50
Wakefulness	5 min = 1 min > 20 s	5 min = 1 min = 20 s	5 min = 1 m	5 min = 1 min = 20 s	5 min = 1 min = 20 s
Drowsiness	5 min = 1 min = 20 s	5 min = 1 min = 20 s	5 min > 1 m	5 min = 1 min ≤ 20 s ^1^	5 min = 1 min = 20 s
Non-REM	5 min = 1 min > 20 s	5 min > 1 min = 20 s	5 min = 1 m	5 min = 1 min = 20 s	5 min = 1 min < 20 s
REM	5 min = 1 min = 20 s	5 min = 1 min = 20 s	5 min = 1 m	5 min = 1 min = 20 s	5 min = 1 min = 20 s

^1^ In case of the rMSSD parameter in drowsiness state, the significant difference was only between the 5 min and the 20 s sample.

**Table 2:** Differences between sleep stages using the various ECG parameters calculated based on the longest period of the given stage. Following false discovery rate adjustment differences with a *p* < 0.01, remain significant at α = 0.05.

**HR**	*Awake*	*Drowsiness*	*NREM*
χ^2^(3) = 19.7; *p* < 0.001			
*Drowsiness*	t_(11)_ = 6.807; *p* < 0.001		
*Non-REM*	t_(11)_ = 6.946; *p* < 0.001	t_(11)_ = 1.773; *p* = 0.104	
*REM*	t_(11)_ = 5.112; *p* <0.001	t_(11)_ = 0.271; *p*=0.792	t_(11)_ = 1.342; *p* = 0.207
**SDRR**	*Awake*	*Drowsiness*	*NREM*
χ^2^_(3)_ = 15.9; *p* < 0.001			
*Drowsiness*	t_(11)_ = 2.675; *p* <0.05		
*Non-REM*	t_(11)_ = 7.476; *p* <0.001	t_(11)_ = 0.370; *p* = 0.718	
*REM*	t_(11)_ = 5.594; *p* <0.001	t_(11)_ = 0.173; *p* = 0.866	t_(11)_ = 1.215; *p* = 0.250
**mSD20secRR**	*Awake*	*Drowsiness*	*NREM*
χ^2^_(3)_ = 15.7; *p* < 0.001			
*Drowsiness*	t_(11)_ = 4.02; *p* < 0.01		
*Non-REM*	t_(11)_ = 3.192; *p* < 0.01	t_(11)_ = 0.116; *p* = 0.91	
*REM*	t_(11)_ = 5.229; *p* < 0.001	t_(11)_ = 1.426; *p* = 0.182	t_(11)_ = 0.959; *p* = 0.358
**RMSSD**	*Awake*	*Drowsiness*	*NREM*
χ^2^_(3)_ = 12.7; *p* < 0.01			
*Drowsiness*	t_(11)_ = 2.833; *p* < 0.05		
*Non-REM*	t_(11)_ = 4.217; *p* = 0.001	t_(11)_ = 1.022; *p* = 0.329	
*REM*	t_(11)_ = 3.668; *p* < 0.01	t_(11)_ = 0.959; *p* = 0.358	t_(11)_ = 0.221; *p* = 0.829
**RR50 count**			
χ^2^_(3)_ = 2.75; *p* = 0.432			
**pRR50**	*Awake*	*Drowsiness*	*NREM*
χ^2^_(3)_ = 11.8; *p* < 0.01			
*Drowsiness*	t_(11)_ = 3.676; *p* < 0.01		
*Non-REM*	t_(11)_ = 4.242; *p* = 0.001	t_(11)_ = 1.608; *p* = 0.136	
*REM*	t_(11)_ = 3.172; *p* < 0.01	t_(11)_ = 0.659; *p* = 0.523	t_(11)_ = 4.037; *p* < 0.01

**Table 3:** Summary of results comparing the different sampling intervals from the first occurrence of all sleep stages. The direction of significant differences are indicated with inequality signs (>, <). Results that deviate from the analysis based on the longest occurrence of the sleep stages are marked with italics.

	HR	SDRR	mSD20sRR	RMSSD	pRR50%
Wakefulness	5 min = 1 min ≥ 20 s ^1^	5 min = 1 min = 20 s	5 min = 1 m	5 min = 1 min = 20 s	5 min = 1 min = 20 s
Drowsiness	5 min < 1 min = 20 s	5 min < 1 min = 20 s	5 min > 1 m	5 min = 1 min = 20 s	5 min = 1 min = 20 s
non-REM	5 min = 1 min = 20 s	5 min > 1 min = 20 s	5 min > 1 m	5 min = 1 min = 20 s	5 min = 1 min = 20 s
REM	5 min = 1 min = 20 s	5 min = 1 min = 20 s	5 min = 1 min	5 min = 1 min = 20 s	5 min = 1 min = 20 s

^1^ In case of the HR parameter in the wakefulness state, there was no significant difference between sampling intervals, but a non-significant trend (*p* < 0.1) indicated the same direction as with the previous sampling method.

**Table 4:** Differences between sleep stages using the various ECG parameters calculated based on the first occurrence of the given stage.

**HR**	*Awake*	*Drowsiness*	*NREM*
χ^2^_(3)_ = 21.9; *p* < 0.001			
*Drowsiness*	t_(11)_ = 5.672; *p* < 0.001		
*Non-REM*	t_(11)_ = 5.394; *p* < 0.001	t_(11)_ = 2.722; *p* = 0.020	
*REM*	t_(11)_ = 5.356; *p* < 0.001	t_(11)_ = 2.593; *p* = 0.025	t_(11)_ = 0.311; *p* = 0.761
**SDRR**	*Awake*	*Drowsiness*	*NREM*
χ^2^_(3)_ = 32.0; *p* < 0.001			
*Drowsiness*	t_(11)_ = 3.751; *p* = 0.003		
*Non-REM*	t_(11)_ = 6.291; *p* < 0.001	t_(11)_ = 4.077; *p* = 0.002	
*REM*	t_(11)_ = 5.884; *p* < 0.001	t_(11)_ = 4.447; *p* = 0.001	t_(11)_ = 2.187; *p* = 0.051
**mSD20secRR**	*Awake*	*Drowsiness*	*NREM*
χ^2^_(3)_ = 22.3; *p* < 0.001			
*Drowsiness*	t_(11)_ = 0.133; *p* = 0.897		
*Non-REM*	t_(11)_ = 0.879; *p* = 0.398	t_(11)_ = 2.747; *p* = 0.019	
*REM*	t_(11)_ = 6.481; *p* < 0.001	t_(11)_ = 2.126; *p* = 0.057	t_(11)_ = 1.548; *p* = 0.150
**RMSSD**	*Awake*	*Drowsiness*	*NREM*
χ^2^_(3)_ = 15.1; *p* = 0.002			
*Drowsiness*	t_(11)_ = 4.037; *p* = 0.002		
*Non-REM*	t_(11)_ = 5.126; *p* < 0.001	t_(11)_ = 2.241; *p* = 0.047	
*REM*	t_(11)_ = 4.420; *p* = 0.001	t_(11)_ = 2.357; *p* = 0.038	t_(11)_ = 0.775; *p* = 0.454
**RR50 count**			
χ^2^_(3)_ = 7.347; *p* = 0.062			
**pRR50%**	*Awake*	*Drowsiness*	*NREM*
χ^2^_(3)_ = 17.7; *p* = 0.001			
*Drowsiness*	t_(11)_ = 4.507; *p* = 0.001		
*Non-REM*	t_(11)_ = 4.117; *p* = 0.002	t_(11)_ = 0.890; *p* = 0.393	
*REM*	t_(11)_ = 4.028; *p* = 0.002	t_(11)_ = 0.434; *p* = 0.673	t_(11)_ = 1.903; *p* = 0.084

Following false discovery rate adjustment differences with a *p* < 0.01, remain significant at α = 0.05.

**Table 5:** Differences between ECG parameters following positive versus negative social interactions (5 min sampling from the first occurrence of a given sleep stage). Significant differences are highlighted with bold.

	Wake	Drowsiness	Non-REM	REM
**HR**	**t_(13)_ = 3.473; *p* = 0.004**	t_(13)_ = 0.888; *p* = 0.391	t_(13)_ = 2.356; *p* = 0.035	t_(13)_ = 0.313; *p* = 0.760
**SDRR**	t_(13)_ = 1.250; *p* = 0.233	t_(13)_ = 1.599; *p* = 0.134	**t_(13)_ = 2.800; *p* = 0.015**	t_(13)_ = 0.165; *p* = 0.872
**mSD20s**	**t_(13)_ = 4.674; *p*<0.001**	t_(13)_ = 1.393; *p* = 0.187	t_(13)_ = 1.166; *p* = 0.264	t_(13)_ = 1.182; *p* = 0.260
**RMSSD**	**t_(13)_ = 4.542; *p* = 0.001**	t_(13)_ = 1.099; *p* = 0.292	t_(13)_ = 2.064; *p* = 0.060	t_(13)_ = 0.052; *p* = 0.960
**RR50c**	**t_(13)_ = 2.556; *p* = 0.024**	t_(13)_ = 0.363; *p* = 0.722	t_(13)_ = 1.359; *p* = 0.197	t_(13)_ = 2.287; *p* = 0.041
**pRR50%**	**t_(13)_ = 4.274; *p* = 0.001**	t_(13)_ = 0.691; *p* = 0.502	t_(13)_ = 0.571; *p* = 0.578	t_(13)_ = 0.101; *p* = 0.922

Following False discovery rate adjustment differences with a *p* < 0.03, remain significant at α = 0.05.

**Table 6:** Differences between ECG parameters following positive versus negative social interactions (5-min sampling from the first occurrence of a given sleep stage). Significant differences are highlighted with bold

	Wake	Drowsiness	Non-REM	REM
**HR**	**r = 0.855, *p* < 0.001**	**r = 0.738, *p* = 0.004**	**r = 0.918, *p* < 0.001**	**r = 0.774, *p* = 0.003**
**SDRR**	r = 0.185, *p* = 0.546	r = 0.309, *p* = 0.304	r = 0.522, *p* = 0.067	**r = 0.649, *p* = 0.022**
**mSD20s**	r = 0.538, *p* = 0.058	r = 0.189, *p* = 0.537	r = 0.357, *p* = 0.232	r = -0.215, *p* = 0.503
**RMSSD**	r = 0.442, *p* = 0.131	r = 0.073, *p* = 0.813	**r = 0.766, *p* = 0.002**	**r = 0.807, *p* = 0.001**
**RR50**	r = 0.533, *p* = 0.061	**r = 0.708, *p* = 0.007**	**r = 0.792, *p* = 0.001**	**r = 0.814, *p* = 0.001**
**pRR50%**	**r = 0.643, *p* = 0.018**	r = -0.107, *p* = 0.728	r = 0.570, *p* = 0.042	r = 0.458, *p* = 0.134

Following False discovery rate adjustment differences with a *p* < 0.03, remain significant at α = 0.05.

## Supplementary Materials

The following are available online at http://www.mdpi.com/2076-2615/8/7/107/s1.

## Abbreviations

REM	Rapid Eye Movement sleep;
Non-REM	Non-Rapid Eye Movement sleep;
EEG	Electroencephalography;
HR	Heart rate;
HRV	Heart rate variability
