# Supplementary material for: Heart Rate and Heart Rate Variability during Sleep in Family Dogs (Canis familiaris). Moderate Effect of Pre-Sleep Emotions
Source: Animals (Basel). 2018 Jul 2;8(7):107. doi: 10.3390/ani8070107 (PMC6071078; doi:10.3390/ani8070107)
Supplement: Supplementary file 1 [file animals-08-00107-s001.pdf]

Canine Polisomnography Method

All subjects participated in a 3-hour-long sleeping occasion (an afternoon napping) following the protocol described in Kis et al. 2014 [1]. The timing of the recording could vary depending on the preferences of the participating dog owners and human subjects, but was restricted to the period between 12 pm and 6 pm as (apart from night time) dogs, similarly to humans, show the highest propensity to sleep during the afternoon [2].

After a 5-10 minutes exploration and familiarization period the owner took place on the mattress placed on the floor and assisted the two experimenters throughout the process of fixing surface attached electrodes onto the dog. The dog was rewarded with food during electrode placement if the owner deemed it necessary; social reinforcement (praise, petting) was used in all cases.

Sleep was monitored by polysomnography (PSG) that allows the parallel recording of several physiological variables – such as neural oscillations (EEG), eye movements (EOG), muscle tone and movements (EMG), heart rhythm (ECG) and respiration patterns – during sleep. The canine PSG technique was developed following the methodology of the human 10-20 system, thus electrodes were placed on anatomical points keeping the relative (rather than the absolute) distance between electrodes constant. Prior to the acceptance of the final design we had conducted pilot studies to find the most efficient setup and placement of the electrodes. When all of our a priori articulated criteria (production of clear and interpretable signal; impedances below 15 kΩ; both NREM and REM phase during the recording interval) were met, our PSG design (**Figure S1**) was accepted and applied identically for all subjects. Surface attached scalp electrodes were placed over the anteroposterior midline of the skull (Fz, Pz) – similarly to [3] – and on the zygomatic arch (*os zygomaticum*) next to the left eye (O) for electroocolugraphy (EOG). The ground electrode (G) was placed on the left *musculus temporalis*, the Pz electrode served as reference. All EEG and EOG electrodes were placed on a bone so artifacts resulting from muscle movements were minimal. Electrodes were placed bilaterally on the *musculus iliocostalis dorsi* for electromyography (EMG) and over the second rib for electrocardiography (ECG). Respiratory movements were also monitored by a respiratory belt attached to the chest. Gold-coated Ag|AgCl electrodes fixed with EC2 Grass Electrode Cream (Grass Technologies, USA) were used for the recordings. Impedances for the EEG electrodes were kept below 15 kΩ.

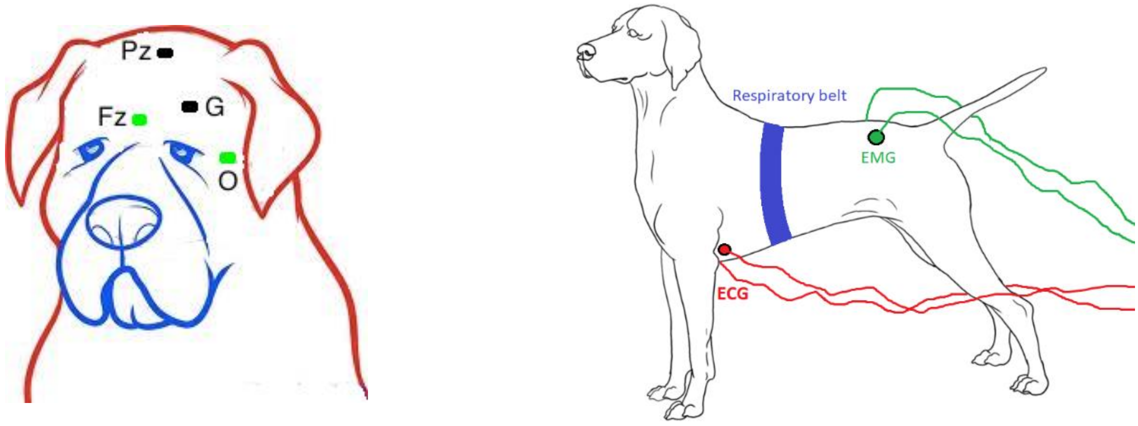

a) **b)**

Figure S1. Electrode placement for the Canine Polisomnography method.
EEG electrodes (a) were placed on then anterior-posterior midline of the skull, with Fz serving as the active electrode and Pz serving as reference. The EOG electrode was placed on the left *os zygomioticum* (O) and referenced to Pz. The ground electrode (G) was placed on the left *musculus temporalis*. Further electrodes (b) were placed on the body of the dog for ECG (second rib bilaterally) and EMG (*musculus iliocostalis dorsi* bilaterally), as well as a respiration belt.

Sleep recordings were visually scored according to standard criteria [4], [5]. The sleep macrostructure of dogs is composed of the stages of awakeness, drowsiness, non-REM and REM (**Figure S2.**). The *wakefulness stage* was defined as the occurrence of fast activity in the EEG (Fz-Pz derivation), high amplitude and frequency eye movements in the EOG (O-Fz derivation), elevated muscle tone and frequent movements (EMG channel). *Drowsiness* was defined as fast EEG activity in the EEG channel (Fz-Pz derivation) accompanied by decreased amplitude and frequency eye movements in the EOG (O-Fz derivation), lowered but observable muscle tone (EMG channel) and fairly regular respiration (Rsp channel). S*low wave sleep* (SWS, Non-REM) was defined as the occurrence of ≥15 μV delta (1-4 Hz) activity and/or sleep spindles (waves with 12-16 Hz frequency and ≥0.5 sec duration) in the EEG (Fz-Pz derivation), no or low amplitude eye movements in the EOG (O-Fz derivation), relatively regular respiration (Rsp channel) and decreased muscle tone (EMG channel). *REM sleep* was defined as the occurrence of rapid eye movements in the EOG (O-Fz derivation) – also seen as artefacts in the EEG (Fz-Pz derivation) –, fast EEG activity (Fz-Pz derivation), muscular atonia (EMG channel), and irregular respiration (Rsp channel). Inter-rater reliability (Cohen's κ) was calculated in previous studies [1], [6], and was found to be "Almost perfect" (κ>0.90) according to the categorization of [7].

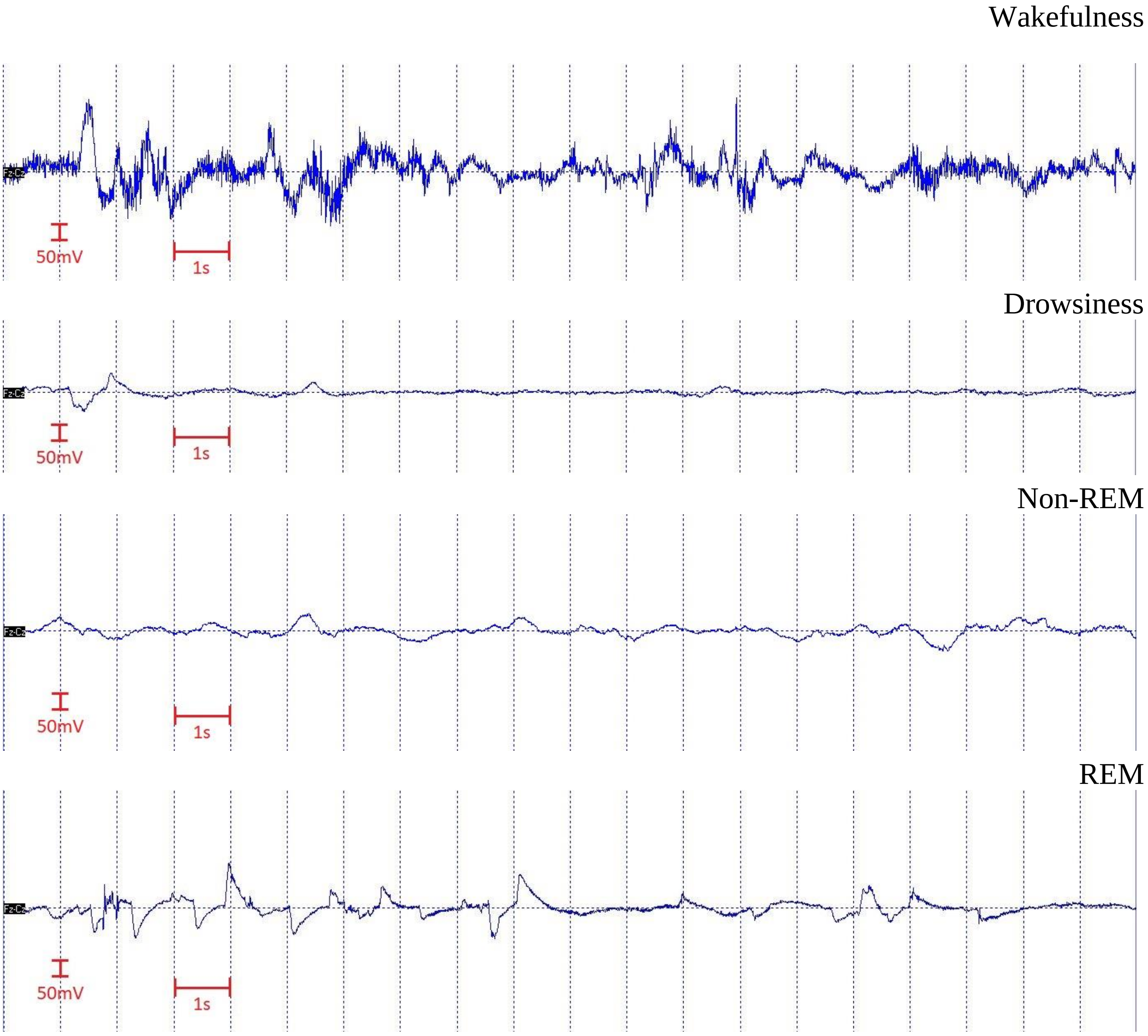

Figure S**2.** EEG pattern of the different stages of dogs' sleep; Fz-Pz derivation.
Beta activity (16–30 Hz) is the dominant characteristic of dogs' awake state. The drowsiness stage is a transition between waking and sleep (characteristic of carnivore or insectivore species where the distinction between the awake and sleeping states is not clear – Zepelin, Siegel, & Tobler, 2005), and has been observed in dogs as well, with the highest power in the alpha band. The subdivision of non-REM into different stages is only used in some primates (Irene Tobler, 1995), but it is not a widely used practice for dogs, although some studies had distinguished between light and deep non-REM sleep in carnivores – e.g. for the cat (Ursin, 1968); or for the dog (Wauquier et al., 1979). During REM sleep, dominating beta and theta activity can be observed in dogs, along with an inconsistent pattern of rapid eye movements.
